# A retrospective cohort study on the impact of finerenone on the hepatic fibrosis indicator FIB-4

**DOI:** 10.3389/fendo.2026.1745102

**Published:** 2026-02-10

**Authors:** Bo An, Fang Chen, Wencheng An, Zhijun Cui, Lin Jiang, Shujing Zhou, Mingda Song, Wei Huang, Huixian Yan

**Affiliations:** Department of Endocrinology, Beijing Haidian Hospital, Beijing, China

**Keywords:** FIB-4, finerenone, hepatic fibrosis, nonalcoholic fatty liver disease, real-world data, selective nonsteroidal mineralocorticoid receptor antagonist

## Abstract

**Objective:**

This study aimed to evaluate the influence of finerenone, a novel nonsteroidal selective mineralocorticoid receptor antagonist (nsMRA), on the hepatic fibrosis indicator FIB-4, as well as its safety profile in hospitalized patients with type 2 diabetes mellitus (T2DM) and chronic kidney disease (CKD) managed in the endocrinology department.

**Methods:**

A single-center, retrospective cohort design was employed, enrolling 138 consecutive hospitalized patients who initiated finerenone therapy for the first time in our endocrinology department between June 2023 and December 2024. Baseline data and subsequent outpatient follow-up records were extracted to calculate the FIB-4 and assess changes in hepatic and renal function, serum potassium levels, and adverse events during treatment.

**Results:**

Of the enrolled cohort, 119 patients (85.6%) had complete baseline laboratory profiles, with a mean age of 63.1 years and 58% male. Baseline FIB-4 median 1.15. Among 52 patients (43%) who completed follow-up, the median follow-up duration was 6 months (range 1–16 months). The median follow-up FIB-4 declined to 1.10, representing an absolute reduction of 0.05. Patients with follow-up exceeding 12 months follow-up showed a further reduction to 0.96. Importantly, no cases of treatment discontinuation due to hyperkalemia were documented during follow-up.

**Conclusion:**

In real-world clinical practice, finerenone in T2DM patients with CKD trended to lower FIB-4 values, especially with longer use. However, no statistical significance and study limitations preclude definitive conclusions on its antifibrotic activity. These findings are hypothesis-generating and need confirmation in large, prospective, long-term studies with robust endpoints and proper controls.

## Introduction

Nonalcoholic fatty liver disease (NAFLD) is currently the most prevalent chronic liver disorder worldwide, affecting approximately 43% of individuals with type 2 diabetes mellitus (T2DM) (1). Complications such as NASH-related cirrhosis, hepatocellular carcinoma (HCC), and acute-on-chronic liver failure constitute major indications for adult liver transplantation (2). Hepatic fibrosis is central to disease progression, and reversal at an early stage can significantly reduce long-term liver-related mortality (3). Despite this, no pharmacological agents have been formally approved for antifibrotic treatment of NAFLD/NASH, underscoring an urgent clinical need for effective interventions.

The hepatic fibrosis indicator FIB-4 (calculated as age × AST/platelets × √ALT) is a simple, cost-effective, and reproducible tool jointly recommended by the American Association for the Study of Liver Diseases (AASLD) and the European Association for the Study of the Liver (EASL) for primary care screening and longitudinal monitoring (4). Variations in the FIB-4 correlate with changes in fibrosis stage (5). In the NHANES cohort in the United States, a 1-unit increase in FIB-4 was positively associated with all-cause mortality and cardiovascular mortality, with hazard ratios of 1.24 and 1.17, respectively (6). Thus, the identification of safe, accessible, and efficacious agents capable of reversing early-stage fibrosis is an area of significant research interest.

The mineralocorticoid receptor (MR) is expressed not only in renal tubules and myocardium but also in hepatocytes, hepatic stellate cells (HSCs), and Kupffer cells (7). Hyperglycemia, hyperaldosteronism, and oxidative stress can activate MR, triggering upregulation of the TGF-β1/Smad, NLRP3 inflammasome, CTGF, and platelet-derived growth factor (PDGF) signaling pathways, which in turn promote HSC activation, collagen synthesis, and fibrous septa formation (8). In animal models, MR gene deletion or administration of mineralocorticoid receptor antagonists (MRAs) markedly reduced α-SMA and COL1A1 expression and improved fibrosis induced by carbon tetrachloride or high-fat diet (9, 10). However, the long-term use of spironolactone is constrained by sex hormone-related adverse effects (e.g., gynecomastia in males, menstrual irregularities in females) and the risk of hyperkalemia.

Finerenone, a third-generation highly selective nsMRA, exhibits MR affinity comparable to spironolactone but with over 500-fold greater selectivity and negligible gonadal side effects (11). Two pivotal phase III clinical trials, FIDELIO-DKD and FIGARO-DKD, enrolling over 13,000 patients with T2DM and CKD, demonstrated that finerenone significantly reduced rates of renal failure, cardiovascular mortality, and hospitalization for heart failure, while maintaining a controllable risk profile for hyperkalemia (12, 13). Subgroup analyses further indicated that in patients with impaired hepatic function, finerenone did not adversely affect liver enzymes and preserved cardiorenal benefits. Moreover, cardiovascular benefits were more pronounced in individuals with higher FIB-4 (14).

Given that patients managed within endocrinology services frequently present with T2DM, NAFLD, and early fibrosis, this study utilized real-world data to retrospectively evaluate dynamic changes in FIB-4 before and after initiation of finerenone in patients with T2DM and CKD, aiming to provide preliminary evidence for potential nsMRA application in hepatic fibrosis intervention.

## 2 Materials and methods

### 2.1 Study design

This investigation was conducted as a single-center, retrospective cohort study, designed to evaluate clinical outcomes within a defined patient population using systematically collected historical data.

### 2.2 Study population

Eligible participants were adults aged ≥18 years diagnosed with type 2 diabetes mellitus (T2DM) and chronic kidney disease (CKD), who were hospitalized in the endocrinology department of our institution between June 2023 and December 2024 and initiated finerenone therapy for the first time. Exclusion criteria included: ① baseline cirrhosis, hepatic carcinoma, or history of liver transplantation; ② presence of other chronic liver diseases such as hepatitis B virus (HBV) infection, hepatitis C virus (HCV) infection, autoimmune hepatitis, or drug-induced liver injury; ③ alcohol consumption exceeding 30 g/day; ④ absence of baseline laboratory data; and ⑤ discontinuation of finerenone for more than 4 weeks during follow-up.

### 2.3 Data collection

Comprehensive clinical and laboratory data were retrieved from the hospital’s electronic medical record (EMR) system and the outpatient follow-up database for analysis.

(1) Demographic variables included age, sex, body mass index (BMI), and duration of T2DM;
(2) Laboratory parameters comprised alanine aminotransferase (ALT), aspartate aminotransferase (AST), platelet count (PLT), glycated hemoglobin (HbA1c), estimated glomerular filtration rate (eGFR; calculated using the CKD-EPI formula), urinary albumin-to-creatinine ratio (UACR), and serum potassium concentration;
(3) Adverse events were recorded, including high potassium blood levels (hyperkalemia, defined as serum potassium >5.5 mmol/L), acute kidney injury (AKI), symptomatic hypotension, and breast pain or enlargement.

### 2.4 Calculation of FIB-4 and grouping

The hepatic fibrosis indicator FIB-4 index was calculated as: FIB-4 = age (years) × AST (U/L)/[platelet count (10^9^/L) × √ALT (U/L)]. Patients were categorized into two groups according to follow-up duration: ≤6 months and >6 months, with an additional stratification at 12 months.

### 2.5 Endpoints and definitions

The primary endpoint was the absolute change in FIB-4 relative to baseline at the conclusion of follow-up.

Secondary endpoints included: ① FIB-4 decreased in patients with a follow-up of more than 6 months or less than 6 months; ② FIB-4 decreased after more than 12 months of follow-up; ③safety outcomes, including hyperkalemia and AKI.

### 2.6 Statistical analysis

Analyses were performed using spss software version 22.0. The Shapiro-Wilk method was employed to test the normality of the measurement data. Measurement data that followed a normal distribution were presented in the form of x ± s, and comparisons between the two groups were performed using the independent sample t-test. Measurement data that did not follow a normal distribution were presented in the form of M (Q1, Q3), and comparisons between before and after initiation(of finerenone) were conducted using the Mann-Whitney U test or Wilcoxon Signed-Rank Test. Count data were expressed as n (%). A two-sided P-value <0.05 was deemed statistically significant.

## 3 Results

### 3.1 Baseline characteristics

Among 138 patients initiating finerenone, 119 (85.6%) had complete baseline datasets. The mean age was 63.1 ± 9.8 years; 69 patients (58%) were male. The mean BMI was 25.7 ± 3.9 kg/m², mean duration of T2DM 12.4 ± 7.2 years, and mean HbA1c 8.1 ± 1.3%. Baseline mean eGFR was 79 ± 21 mL·min^−1^·1.73 m^−2^, and median UACR was 240.6 mg/g. The baseline median FIB-4 index was 1.15(0.85,1.68), with 48 patients (40.3%) having FIB-4 ≥1.3, and 15 patients (12.6%) having FIB-4 ≥2.67 (**Table 1**).

**Table 1:** Baseline data of hospitalized patients.

	N=138 (Initiated finerenone during hospitalization)	N=119 (Initiated finerenone during hospitalization and had baseline FIB-4 data)
Mean age (years)	62.7 ± 13.24	63.1 ± 12.83
Male proportion (%) (n)	57.2(79)	58(69)
Duration of T2DM (years)	12.1 ± 7.9	12.4 ± 7.2
Mean BMI (kg/m²)	-	25.7 ± 3.9
HbA1c (%)	-	8.1 ± 1.3
Baseline eGFR (ml·min^−1^·1.73m²)	79.8± 23.1	78.9 ± 24.4
Median UACR (mg/g)	250.3	240.6
Median FIB-4	-	1.15(0.85,1.68)
Number (proportion) of patients with FIB-4 ≥1.3	-	48(40.3%)
Number (proportion) of patients with FIB-4≥2.67	-	15(12.6%)

### 3.2 Follow-up completion

Follow-up in the outpatient setting was completed by 52 patients post-discharge, with a median follow-up duration of 6 months (range: 1–16 months). Loss to follow-up was attributed to transfer to other facilities (n=45), refusal to attend follow-up (n=27), death (n=3), and inability to establish contact (n=11).

### 3.3 Dynamic changes in FIB-4

At the end of follow-up, the median FIB-4 decreased to 1.10(0.74,1.65), representing an absolute reduction of 0.05 (P = 0.15).

Stratified analysis:

(1) Patients with follow-up <6 months (n=26) exhibited a median FIB-4 of 1.15, with no statistically significant change compared with baseline (P = 0.17);
(2) Patients with follow-up ≥ 6 months (n=26) had a median FIB-4 of 1.10, showing a reduction of 0.05 from baseline (P = 0.14);
(3) Patients followed for ≥12 months (n=12) demonstrated a further decline in median FIB-4 to 0.96, a reduction of 0.19 from baseline (P = 0.11) (**Table 2**).
(4) For patients with baseline FIB-4 ≥ 1.3, 17 patients underwent follow-up visits. The FIB-4 data of these 17 patients were compared before and after treatment. The median baseline FIB-4 value was 2.41, and the FIB-4 value at the follow-up visit was 1.95 (with a median follow-up period of 5 months), (P = 0.036) (**Table 3**).

**Table 2:** Changes in FIB-4.

	Initiated finerenone during hospitalization (N = 138)	Initiated finerenone during hospitalization and had baseline FIB-4 data (N = 119)	Patients reexamined in the outpatient clinic (N = 52)
Age (years)	62.7 ± 13.24	63.1 ± 12.83	61.5 ± 14.6
Male proportion (%)	50	58	58
Mean eGFR(ml/min*1.73m²)	79.8± 23.1	78.9 ± 24.4	71.7
Median UACR(mg/g)	-	240.61	185
Median follow-up time (months)	-	-	6
FIB-4 Follow-up ≥6 months Follow-up <6 months Follow-up ≥12 months Follow-up <12 months	-	1.15(0.85,1.68)	1.10(0.74,1.65) 1.10(0.88,1.58) 1.15(0.73,1.93) 0.96(0.69,1.58) 1.14(0.76,1.74)

**Table 3:** The change of FIB-4 in patients with baseline FIB-4 ≥ 1.3.

	Baseline (N = 17)	Follow up (N = 17)	P value
Age (years)	69.7	69.7	-
Male proportion (%)	53	53	-
Mean eGFR(ml/min*1.73m²)	62.5 ± 4.9	60.0 ± 4.8	-
Median follow-up time (months)	-	5(3,10)	-
FIB-4(median)	2.41(1.75,3.12)	1.95(1.58,3.01)	0.036*

*Wilcoxon SignedRank Test.

### 3.4 Safety

Over the follow-up period, mean serum potassium increased from 4.1 ± 0.4 mmol/L to 4.3 ± 0.5 mmol/L. Hyperkalemia (>5.5 mmol/L) occurred in 2 patients (3.8%), both of whom achieved normalization following finerenone dose reduction and dietary potassium restriction. No cases of AKI were observed, and no reports of breast tenderness/enlargement, menstrual irregularities, or hypotensive syncope were recorded.

## 4 Discussion

This investigation represents the first real-world clinical observation linking finerenone therapy with a downtrend in the hepatic fibrosis indicator FIB-4 among patients with T2DM and CKD, with the magnitude of benefit appearing to be time-dependent. These findings provide preliminary clinical evidence might support the potential role of nonsteroidal mineralocorticoid receptor antagonists (nsMRAs) in modulating hepatic fibrosis, while underscoring the need for further validation.

### 4.1 The extrapolation of the anti-inflammatory and antifibrotic mechanisms of finerenone: from kidney to liver

Finerenone exerts its pharmacologic effects through selective blockade of the mineralocorticoid receptor (MR), thereby inhibiting NF-κB, TGF-β1/Smad, and SGK1 signaling pathways. This leads to reduced secretion of pro-inflammatory cytokines such as interleukin-6 (IL-6) and connective tissue growth factor (CTGF), and attenuation of collagen deposition (15). Hepatic stellate cells (HSCs) express MR abundantly, and activation of the aldosterone–MR pathway promotes HSC transdifferentiation into myofibroblasts, a central event in hepatic fibrogenesis (16). Although prospective clinical trials on finerenone for hepatic fibrosis are lacking, the 2024 FIDELITY liver subgroup analysis (n=13,026) revealed that in patients with CKD and T2DM, finerenone exerted a neutral effect on liver enzyme levels (ALT, AST, gamma-glutamyl transferase [GGT]), without inducing hepatotoxicity. Notably, higher FIB-4 values correlated with increased cardiovascular event rates, and finerenone reduced the composite cardiovascular risk by 52% even in the high FIB-4 subgroup (>3.25) (14). Given the presence of metabolic characteristics such as insulin resistance and oxidative stress in the study population, finerenone may synergistically inhibit the activation of hepatic stellate cells by improving metabolic inflammation and reducing oxidative stress injury. This suggests a multitargeted protective role of MR blockade in metabolic-associated fatty liver disease. The observed trend of decreased FIB-4 with prolonged therapy in our cohort aligns with this clinical signal, may indirectly supporting the hypothesis that MR blockade may confer antifibrotic effects beyond the kidney.

### 4.2 Clinical significance of dynamic changes in FIB-4

A FIB-4 value <1.45 has been validated as a reliable threshold to exclude significant hepatic fibrosis (METAVIR stage F≥2), with a negative predictive value exceeding 90% (17). In a study of 911 patients with hepatitis B-related cirrhosis, a ≥1.0 decrease in FIB-4 within one year was associated with a markedly reduced risk of hepatocellular carcinoma (hazard ratio ≈0.55) (18). However, analogous data for patients with nonalcoholic fatty liver disease (NAFLD) are currently unavailable. In our study cohort of T2DM and CKD patients, finerenone therapy was associated with a median FIB-4 reduction of 0.05 overall, and 0.19 in the subgroup followed for ≥12 months, suggesting a favorable, albeit modest, effect on hepatic fibrosis indicators. Among patients with baseline FIB-4 ≥1.3, the changes in FIB-4 were statistically significant. This might indicate that for patients with higher FIB-4 values, they might benefit earlier from the treatment with finerenone.

### 4.3 Time-effect relationship and clinical implications

Subgroup analysis demonstrated that patients with follow-up <6 months showed negligible change from baseline, whereas reductions were observed in those followed for≥6 months, with further decline at ≥12 months. This temporal response mirrors findings from phase III finerenone trials, wherein after 16 months of continuous therapy, the eGFR decline slope in the treatment group crossed that of the placebo group, indicating time-dependent renal benefit (12, 13). Clinically, FIB-4 offers a cost-effective, reproducible means for longitudinal monitoring in high-risk populations with metabolic syndrome and NAFLD.

### 4.4 Study limitations and future directions

The study’s retrospective, single-arm design and limited sample size constrain generalizability and introduce potential biases. The absence of a matched control group precludes definitive attribution of FIB-4 changes to finerenone alone, as spontaneous disease improvement or concomitant medications could have contributed to the observed effects. Additionally, the lack of confirmatory liver histology or transient elastography (e.g., FibroScan) means that the reduction in FIB-4 cannot be directly correlated with histological improvements in hepatic fibrosis. The concurrent use of agents such as SGLT2 inhibitors (SGLT2i) and glucagon-like peptide-1 receptor agonists (GLP-1RA), especial their combination treatment significantly improved liver dysfunction and prevented the progression of FIB‐4 index category among patients with an FIB‐4 index ≥1.3 (19), introduces potential confounding that was not accounted for through stratification or multivariate adjustment in our analysis. The lack of statistical significance in FIB-4 reduction is likely due to the short follow-up duration, low completion rate (only 43% of patients with baseline FIB-4 data completed the follow-up), and limited statistical power. These limitations collectively undermine the robustness of our findings and highlight the need for more rigorous study designs. Future investigations should employ multicenter, prospective, randomized controlled trials with FibroScan or MRI-proton density fat fraction (PDFF) as primary endpoints to provide more direct assessments of hepatic fibrosis. Follow-up durations should exceed 12 months to capture the long-term effects of finerenone. Additionally, incorporating advanced molecular techniques such as single-cell sequencing and mass cytometry may elucidate the complex interplay between mineralocorticoid receptor (MR) blockade, hepatic stellate cell subpopulations, and immune cell dynamics, guiding the development of integrated “liver–kidney–heart” antifibrotic strategies.

## 5 Conclusion

In real-world clinical settings, finerenone therapy in patients with T2DM and CKD was associated with a trend towards a reduction in FIB-4 values, particularly over longer treatment durations. However, the lack of statistical significance and the methodological limitations of our study preclude definitive conclusions regarding finerenone’s antifibrotic activity. These findings should be considered hypothesis-generating and warrant confirmation in large-scale, prospective, long-term studies with robust endpoints and appropriate controls.

## Data Availability

The raw data supporting the conclusions of this article will be made available by the authors, without undue reservation.
